# A landscape review with novel criteria to evaluate microbial drivers for cancer: priorities for innovative research targeting excessive cancer mortality in sub-Saharan Africa

**DOI:** 10.3389/fcimb.2025.1625818

**Published:** 2025-08-20

**Authors:** Rebecca Toumi van Dorsten, Robert F. Breiman

**Affiliations:** ^1^ Infectious Diseases and Oncology Research Institute, Faculty of Health Sciences, University of the Witwatersrand, Johannesburg, South Africa; ^2^ South African Medical Research Council Wits Antiviral Gene Therapy Research Unit, Faculty of Health Sciences, University of the Witwatersrand, Johannnesburg, South Africa; ^3^ South African Medical Research Council Vaccines and Infectious Diseases Analytics Research Unit, School of Pathology, Faculty of Health Sciences, University of the Witwatersrand, Johannnesburg, South Africa; ^4^ Department of Global Health, Rollins School of Public Health, Emory University, Atlanta, GA, United States

**Keywords:** infectious disease, criteria for microbial oncogenesis, cancer causation, sub-Saharan Africa (SSA), oncovirus and cancer, infectious disease and cancer, microbiome and cancer

## Abstract

The escalating cancer burden in Sub-Saharan Africa (SSA), with projected doubling of incidence and mortality by 2040, necessitates innovative, cost-effective strategies for prevention, diagnosis, and treatment. While known infectious triggers like HPV, hepatitis viruses, and *H. pylori* account for an estimated 28.7% of cancers in SSA, the full scope of microbially-mediated oncogenesis remains underexplored. We examine existing data and formulate plausible hypotheses regarding the potential roles of additional infectious agents in cancer development within SSA. We explore mechanisms through which microbes may directly or indirectly contribute to oncogenesis, including the action of viral oncogenes, induction of chronic inflammation, mutational signatures, and the impact of immunosuppression, particularly in the context of HIV. Potential microbial triggers warrant further investigation, such as viruses (MMTV, CMV, polyomaviruses, SARS-CoV-2), bacteria (*Fusobacterium nucleatum, Cutibacterium acnes, Salmonella Typhi*), fungi (*Candida, Aspergillus*), parasites (*Schistosoma japonicu*m and *mansoni* and *Toxoplasma gondii*) and the complex interplay with the microbiome. Given the significant challenges in establishing causation for microbial facilitators of cancer, with traditional postulates showing limited utility, we propose a refined set of criteria tailored to microbial oncogenesis, aiming to guide future research efforts. These criteria incorporate elements of both Koch’s postulates and the Bradford Hill framework, adapted to address the unique characteristics of microbial interactions with human hosts. By leveraging existing knowledge and plausible causal relationships, and by implementing advanced experimental tools such as next-generation sequencing and multi-omics analyses, coupled with machine learning approaches and collaborative, multidisciplinary research, we propose to accelerate the identification of novel microbial links to cancer. This knowledge may pave the way for targeted interventions such as new approaches for screening and diagnosis, and strategies for prevention including vaccine development or modification of existing vaccines (or recommendations for immunization timing and population targets). While acknowledging the inherent complexities of studying polymicrobial interactions and the challenges of translating *in vitro* findings to human populations, this work aims to provide a framework for future research and intervention strategies to reduce the escalating cancer burden and address global inequities in SSA. The ultimate goal is to inform evidence-based public health policies and clinical practices that will improve cancer outcomes in this vulnerable region.

## Introduction

Cancer incidence and mortality in Sub Saharan Africa are predicted to double by 2040 (Sharma et al., 2022). In 2020, annual cancer incidence in Sub Saharan Africa (SSA) was estimated at 132 per 100,000 population (age standardized incidence rates) with a mortality rate of 88.9 per 100,000 (Sharma et al., 2022). This equates to 1.1 million new cases and 711,000 deaths from cancer in 2020. The significant prevalence of HIV infection also leads to a higher cancer burden in many parts of SSA with both increased incidence and mortality observed (Casper et al., 2017; Yarchoan and Uldrick, 2018). The rising burden of cancer is coupled with suboptimal access to health care; diagnosis often comes too late for effective treatment or after death (Akuoko et al., 2017; Espina et al., 2017; Mwamba et al., 2023). When cancers are diagnosed early, potentially life-saving therapeutics are frequently not available and access to care, especially in rural and other impoverished, remote areas, is limited with trained oncologists in very short supply (Grover et al., 2015; Ruff et al., 2016; Mallum et al., 2024). Advances in chemotherapy and radiation therapy in high income countries have markedly improved five-year survival for many cancers (Siegel et al., 2021), but new immunotherapies and chemotherapeutic drugs are extremely costly, making them currently out of reach for the vast majority of people living in SSA. Consequently, disparities in cancer burden are increasing. While efforts are underway to bring immunotherapies and advance precision medicine in Africa (Ruff et al., 2016), solutions have not yet been identified to reduce costs and provide access to most Africans. This drives a search for discoveries that could be translated to low-cost, highly effective preventive, diagnostic and therapeutic approaches.

One avenue for advancing cancer prevention, diagnosis and screening, and therapy is to study microbial precipitators for cancer. While not a mainstream cancer research focus, there are dramatic examples of how understanding microbial links to cancers can be transformative. For instance, determining that >90% of cervical cancer cases, the top cause of cancer-related mortality among women in Africa, is precipitated by infection with human papillomavirus (HPV), led to the development and use of highly effective HPV vaccines, which are essentially cancer vaccines (Chan et al., 2019; Lei et al., 2020). It is now possible to prevent cervical cancer in Brazzaville just as effectively as in San Francisco. However, there are still knowledge gaps to fill regarding characteristics and scope of oncogenicity of HPV; for instance, several prevalent oncogenic HPV serotypes (such as 35, 52 and 58) circulating in Africa are not included in the bivalent vaccines currently used for national immunization programs in SSA (Dehlendorff et al., 2021). HIV and HPV are also intrinsically linked, where infection with one, increases infection rates for the other. Moreover, increased cancer progression rates of HPV in the context of HIV infection are significant (Liu et al., 2018; Marima et al., 2021). Vaccine formulations with higher valency will be required to optimize prevention of HPV and subsequently cervical cancer. Optimized screening tests, including self-testing, for detecting HPV in vaginal secretions are also advancing the capacity to save lives through early detection and treatment of cervical cancer (Chan et al., 2019; WHO, 2022). And, importantly, HPV has been shown to cause other cancers, including those that occur in men; yet, currently, the vaccine is routinely given to only girls and not boys, throughout most of Africa.

The effectiveness of prevention of hepatocellular cancer via hepatitis B immunization provides additional evidence for the value of filling knowledge gaps on microbial facilitation of cancers. Vaccines against hepatitis B virus and effective therapeutics against hepatitis C have made attainable the prevention of the vast majority of hepatocellular cancers that are not solely related to chronic alcohol use with attendant cirrhosis (Levrero and Zucman-Rossi, 2016; Virzì et al., 2018; Hwang et al., 2019; Flores et al., 2022).

**Figure 1:**
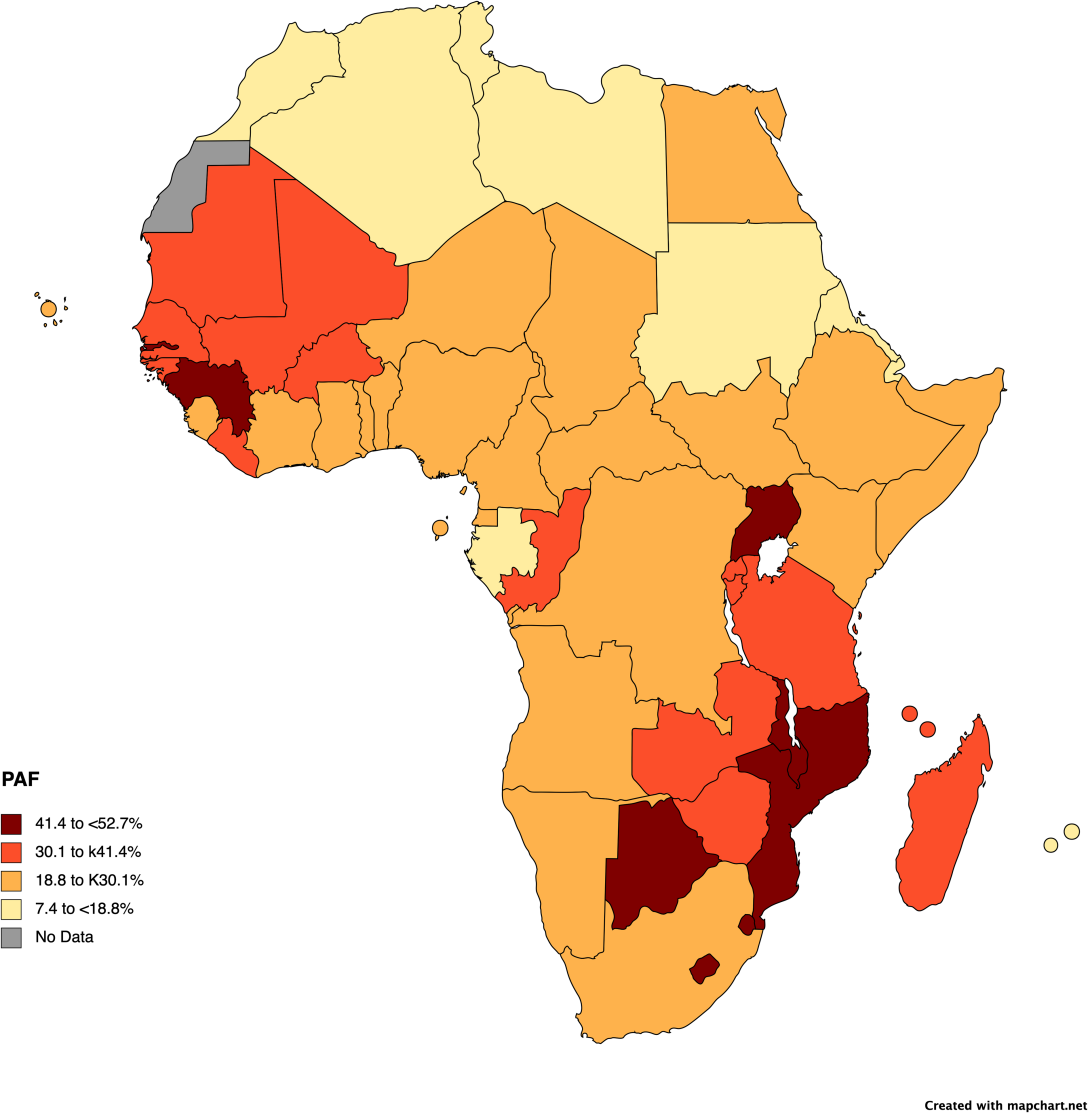
Pathogen attributable fractions of cancer incidence in Africa by country. The map was created with mapchart, using data from the International Agency for Research on Cancer (IARC). Cancers due to HPV, HBV, HCV, and *H. pylori* in 2018 provide the bases for the calculations. Full details for pathogen attributable fraction calculations and data can be found in the following references and within a tool available (and referenced) on the IARC website (de Martel et al., 2020; IARC, 2020; Ngwa et al., 2022).

It is estimated that 28.7% (range: 18-53%; **Figure 1**) of cancers occurring in sub-Saharan Africa are linked to a known infectious trigger (Plummer et al., 2016; de Martel et al., 2020) with the main contributor in this estimate being HPV-related cancers (15% of cancers ranging from 10-38.3%) (**Figure 1**), including oral and throat, penile, and anal cancers in addition to cervical cancer, and HBV HCV, contributing to hepatocellular carcinoma. In addition, some lymphomas (Epstein-Barr virus) and head and neck cancers (Epstein-Barr virus and HPV), gastric cancers (*Helicobacter pylori*), bladder cancers (*Schistosoma haemotobium*), and Kaposi’s sarcoma (Human Herpesvirus 8—HHV8, also referred to as Kaposi’s Sarcoma Herpesvirus-KSHV), have known infectious mediators; however, interventions have not been developed or are not widely used for these other facilitators for cancer, thus far. Prevalence rates for some of these infectious triggers are extremely high with H pylori prevalence ranging to 50-70% in SSA or HPV prevalence similarly showing reports up to 64% (Mbulawa et al., 2018; de Martel et al., 2020; Asempah, 2021; Emmanuel et al., 2024). However, for many cancers, there is lack of systematic surveillance in SSA; thus, prevalence is likely substantially underestimated (Omotoso et al., 2023). The pathogen attributable proportion of cancers will likely become substantially higher than estimated, as new links between microbes and oncogenesis, the focus of this paper, are still being elucidated (de Martel et al., 2020; Ngwa et al., 2022).

In this review, we focus on plausible hypotheses to suggest pathways for additional microbially mediated cancers. Recognizing the immense potential to modify cancer disease burden through the understanding of the role of microbial factors in triggering cancer and/or facilitating its spread and translating that knowledge to new products and/or strategies, we review available data on cancers for which evidence suggests that there may be infectious mediators. Beyond the scope of this paper (but a worthwhile exercise) is detailing the many gaps in using existing knowledge of infection-cancer relationships to develop tools (or optimally use existing tools) or strategies for prevention of cancers; for instance, the demonstrated relationship between *H. pylori* and gastric cancer (Parsonnet et al., 1991) has not led to routinely available approaches to prevent that cancer, nor has the known association of Epstein-Barr virus with Burkitt and Hodgkin lymphoma, and nasopharyngeal carcinoma (Pathmanathan et al., 1995; Brady et al., 2007; Carbone and Gloghini, 2018; Su et al., 2023). Our intent here is to provide a starting point to identify knowledge gaps and define priorities for research on novel infectious mechanisms for oncogenesis.

## How infections cause cancer

### Viral oncogenes and proteins

Pathway alterations and expression of viral oncoproteins are observed in multiple viruses that are directly carcinogenic (Burd, 2003; Young and Murray, 2003; Levrero and Zucman-Rossi, 2016; Kuss-Duerkop et al., 2018; Virzì et al., 2018; Afzal et al., 2022; Burton and Gewurz, 2022; Marongiu et al., 2022). While other unidentified processes are likely, understanding known mechanisms may be helpful for discovering previously unrecognized microbial facilitators of oncogenesis. For example, HPV oncogenesis is mediated by viral oncogenes, such as E6 (Oyervides-Muñoz et al., 2018); E6 recruits intracellular E3 ubiquitin ligase (also named E6AP), which targets p53 for proteasomal degradation (Li et al., 2019). P53 is a tumor suppressor gene which plays critical roles in pathways to prevent DNA damage, marking cells for apoptosis or delaying cell cycle progression in the presence of DNA damage. HPV infection, therefore, inactivates p53 and leads to unregulated cell division, cell growth, cell survival, and DNA damage promotion. E7, another HPV oncogene, also interacts with the p53 pathway through retinoblastoma tumor suppressor protein (pRb), resulting in unregulated cell cycle progression. These oncogenes/proteins are also known to drive other oncogenic pathways, including telomerase regulation. The cancer cells depend on constitutive expression of these proteins, making them prime targets for therapeutic vaccines and biomarker detection (Burd, 2003; Ghebre et al., 2017; Marima et al., 2021).

Oncogene expression tends to interfere with important cell regulation or immortalization pathways. These can also be mediated by host-microbe interactions through proteins which mimic host proteins and interact with key signal pathways causing the overexpression of oncogenes and suppression of tumor suppressor proteins (Guven-Maiorov et al., 2019; Tempera and Lieberman, 2021). For example, EBV produces peptides (BHRF1 and BALF-1) and microRNAs which inhibit Bcl-2 and prevent apoptosis (Young and Murray, 2003; Brady et al., 2007; Carbone and Gloghini, 2018; Burton and Gewurz, 2022). Similarly, KSHV (HHV8) (for which seroprevalence ranges between 30-90% in SSA) inhibits ORF16 which is required for apoptosis (Wan et al., 2024). Viral proteins may also act synergistically with existing cancers by inducing the Warburg effect, i.e. where cells switch to glycolysis fermentation to generate energy instead of oxidative phosphorylation, allowing for the usage of alternative metabolites and the switching to anaerobic respiration (Liberti and Locasale, 2016). For example, HPV, KSHV and Merkel cell polyomavirus (MCPyV) affect glycolysis and induce increased glucose utilization in cancer cells, resulting in stress in healthy surrounding cells. EBV infection can modify lipid and cholesterol metabolism to induce anaerobic metabolism (Burton and Gewurz, 2022). Further mechanisms are reviewed elsewhere (Tempera and Lieberman, 2021)

Direct integration of viruses can cause differential expression of key proteins affecting the cell cycle, DNA repair mechanisms, and apoptosis, each important for oncogenesis. For example, HTLV-1 integrates into CD4+ T-cells causing chromosomal instability, mediated by its oncoprotein RNF8 (Zhi et al., 2020). Integration of HBV can both cause genome instability, and also leads to chronic inflammation due to sustained presence of HBV and its antigens (Borgia et al., 2021; Jin et al., 2023). For HPV and MCPyV, integration into epithelial cells (Yang and You, 2022; Karimzadeh et al., 2023), allows for cell immortalization of these cells due to the constant presence of the oncogenic driver (Elkhalifa et al., 2023).

### Indirect and undefined mechanisms

Pathophysiology of some infectious diseases overlap with oncogenic mechanisms in ways which could promote cancer initiation and growth. Infection with HBV and HCV, for instance, are associated with chronic infection which may lead to chronic inflammation, thereby causing type 2 carcinogenic effects (Borgia et al., 2021; Yang et al., 2021). These viruses are synergistically carcinogenic with other factors such as aflatoxins and liver cirrhosis due to alcohol usage (Borgia et al., 2021; Jin et al., 2023). The links between HBV and HCV and hepatocellular carcinoma are well established, but chronic HBV and HCV infections also may be associated with other cancers, especially gastric adenocarcinomas. For instance, a metanalysis of ten studies found that patients with HBV infection had a higher risk (hazard risk =1.26;95% CI=1.08-1.47)) for gastric cancer when compared with controls (without HBV infection) (Chen et al., 2019; Yang et al., 2021; Yu et al., 2023b). Hypothesized mechanisms awaiting confirmation include chronic inflammation resulting in carcinogenesis, direct viral integration into gastric epithelial cells, expression of viral proteins (like HBx which interferes with cell signaling pathways, gene expression and apoptosis) and immune response modulation (Yang et al., 2021). Likewise, EBV may be associated with a subset of gastric adenocarcinoma; chronic inflammation is one hypothesized mechanism (Salnikov et al., 2024).

### Immunosuppression

HIV causes cancer both by its integration into oncogenes, and, also through an indirect mechanism of immune suppression, which allows existing cancers to progress or oncoviruses to establish infection. HIV/AIDS progression is accompanied by AIDS-defining cancers, such as Non-Hodgkin lymphoma (NHL), Kaposi sarcoma, and cervical cancer) (Beral et al., 1990; Martínez-Maza and Breen, 2002; De Martel et al., 2015; Bohlius et al., 2016; Yarchoan and Uldrick, 2018), with cancer risk in people living with HIV (PLWHIV) significantly elevated, ranging between 25-40%, despite widely accessed antiretroviral therapy (De Martel et al., 2015). Lung cancer, Hodgkin lymphoma, hepatocellular cancer, and anal cancers are associated with HIV infection despite effective ART treatment, suggesting a possible direct oncogenic effect of HIV beyond immune suppression (Khandwala et al., 2021; Navarro et al., 2021; Haas et al., 2022; McGee-Avila et al., 2024). For Burkitt lymphoma, HIV may directly drive oncogenesis through its immunomodulation and engagement of C-C motif chemokine receptor 5 (CCR5) (Samson et al., 1996; Silverberg et al., 2007; Bohlius et al., 2009; Martorelli et al., 2015; Shindiapina et al., 2020). HIV appears to potentiate the oncogenic effect of some viruses to increase risk for cancer. For example, the attributable fraction of EBV-associated Hodgkin lymphoma in the general population is 20-50%, but in HIV-infected patients, 75%-100% of Hodgkin lymphoma is attributable to EBV, possibly due to aberrant CD4 T-cell responses to EBV infection (Carbone and Gloghini, 2018; Shindiapina et al., 2020; Navarro et al., 2021).

Other types of immunosuppression are similarly linked to cancer progression (Haas, 2019; Herrera et al., 2019); for example, advanced age leading to immune senescence and immune suppression drugs are both highly associated with cancer and cancer progression (Haas, 2019; Fu et al., 2023). In addition, tumors facilitate their growth and metastasis by actively suppressing the immune system in their direct microenvironment (Arner and Rathmell, 2023; Tie et al., 2022).

## Criteria for causation

Proving that a microbe facilitates cancer is not straightforward and may vary by pathogen. The Bradford Hill criteria for causation provide epidemiologic evidence for a causal relationship between an exposure and cancer (Bradford Hill, 1965; **Table 1**). The criteria were originally developed to examine environmental exposure (like tobacco smoke, dyes, and other chemicals), but they fall short when assessing potential microbial facilitators of cancer, since microbes, as living organisms, interact with humans in dynamic ways that are in contrast with human interactions with static substances (**Table 1B**). Likewise, Koch’s postulates can be useful for establishing the cause of a novel acute illness due to an infectious disease, but is less relevant for diseases for which there are long delays between exposure and disease expression

**Table 1:** Existing approaches for establishing causation of disease.

1A. Bradford Hill criteria for causation Designed to assess whether environmental exposures or ingestions are associated with specific diseases
1. Effect size (strength of association)
2. Reproducibility (consistency)
3. Specificity (between a factor and effect)
4. Temporality (effect has to occur after the exposure and after an expected delay between cause and effect for cancer)
5. Dose-response relationship (biological gradient)
6. Plausibility (there should be a plausible mechanism for cause and effect although this might be affected by state of current knowledge)
7. Coherence between epidemiologic and laboratory findings
8. Experimental evidence
9. Analogy (between observed association and other associations)
10. Deletion or reversibility effect (if the exposure is deleted then the effect should be reduced or deleted)

1B Koch’s postulates Designed to identify microbial causes for acute infectious diseases of unknown cause
1. Microbe should be found in abundance in all organisms suffering from the disease but not found in healthy organisms (not accounted for in chronic infection or carriage)
2. Microorganism must be isolated from a diseased organism and grown in pure culture (not relevant for microbes that do not grow in pure culture and not relevant for viruses that utilize host cells for growth)
3. The cultured microorganism should cause disease when introduced into a healthy organism
4. The microorganism must be re-isolated from the inoculated, diseased experimental host and identified as identical with the original specific causative agent.

Criteria as per Bradford-Hill’s and Koch’s original criteria (Bradford Hill, 1965; Fedak et al., 2015; Opal, 2017).

Given that study of microbial facilitators for cancer is an emerging discipline, the absence of relevant causation criteria impedes research focus and advances. Adapting and building upon Bradford Hill criteria and Koch’s postulates, we propose a set of criteria for hypothesis generation, to guide study, and to confirm specific microbial oncogenesis (**Table 2**).

**Table 2:** Proposed microbial oncogenesis criteria incorporating Koch’s postulates and Bradford-Hill criteria.

Criteria	Definition/details	Tools		
1. Epidemiologic Association (**)	*Consistent association of a microbe (or a combination of microbes) with a* sp*ecific cancer type within a population (considering demographics and host factors) of humans (or across all populations), especially when compared to people within the same population(s) without cancer (* Mason et al., 2021 *)*	Case-control and Cohort (retrospective and longitudinal) studies Histopathology, immunochemistry, PCR, Genomics and mass spectrometry *Strong association when consistent findings* across different geographical regions and c*onsistent risk* ratios in case-control or cohort studies		
2. Histopathologic association (**)	*Consistent detection of the microbe (virus, bacterium, fungus, or parasite) in cancer tissues compared to healthy controls (* Ziegler et al., 2021 *)*	*In Vitro* assays (PCR, FACS, imaging) Histopathology immunochemistry, PCR, Genomics and mass spectrometry		
3. Temporal association (**)	*Evidence that infection precedes onset of cancer (* Bosch et al., 1995 *;* Walboomers et al., 1999 *)*	Longitudinal (long term) studies		
4. Experimental evidence of facilitation of oncogenesis (**/***) +	*Induction of cancer/precancerous changes upon introduction of the isolated microbe into appropriate models. Does oncogenic cellular transformation occur when a microbe is introduced into an animal (ideally a primate or other human-representative) model or tissue mode (* Ziegler et al., 2021 *).*	3D biosystems (organoids) or animal models		
5. Molecular and Multi-omics evidence for interaction (***)	•Epigenetics (e.g. DNA methylation changes in H. pylori or HPV E6 oncogene resulting in P53 degradation), as well as mutational signatures •Mutation effects (stimulating cellular mutations promoting cancer) •Stimulating immune evasion mechanisms reducing immunosurveillance for emerging cancer cells *Integration of microbial DNA into host genome*	*in vitro* assays, organoids, multi-omics assessments including:		
*a. Molecular Evidence*	*For bacteria*: Identification of toxins, effector proteins, or metabolites that alter cellular signaling *For viruses:* Characterization of viral oncoproteins or insertional mutagenesis *For fungi*: Demonstration of mycotoxins or immune-modulating molecules with carcinogenic potential *For parasites*: Identification of chronic inflammatory responses or direct tissue damage mechanisms Evidence of microbial interference with DNA repair, cell cycle regulation, apoptosis, or immune surveillance (Dyson et al., 1989; Franco et al., 2005; Botelho et al., 2009; Zhu et al., 2021)	
*b. Genomic evidence:* * c. Transcriptomic evidence* * d. Proteomic evidence* * e. Metabolomic evidence* * f. Microbiome analyses*	• Microbial DNA sequences or integration sites in tumor genome; • Host genetic susceptibility factors that enhance oncogenic potential of specific microbes, including demonstration of how microbial exposure can alter (or promote) known host genetic risk factors for cancer. • Demonstratable facilitation of cancer-associated mutational signatures (Péneau et al., 2022) Expression of microbial genes or altered host gene expression profiles (Ego et al., 2005) Detection of microbial proteins or altered host protein responses (Dyson et al., 1989; Celegato et al., 2022) Microbial metabolites or altered host metabolic pathways (Pérez Escriva et al., 2025) Consistent dysbiosis patterns associated with specific cancers (Guo et al., 2021; Pourali et al., 2024)	
6. Prevention (***)	*Does preventing the infection (or removing exposure to the microbe) reduce cancer or evidence of oncogenesis (* Fukase et al., 2008 *;* Yoon et al., 2014 *)*	Clinical trials Relevant animal models	
	*For viruses:* Reduced cancer incidence following vaccination (e.g., HPV, HBV) *For bacteria:* Cancer prevention through antibiotic treatment or bacterial elimination *For fungi:* Antifungal intervention effects on precancerous lesions *For parasites:* Impact of antiparasitic treatment on cancer development *Prevention trials showing reduced cancer incidence after targeting the microbe*	
7. Dose Response relationship (**)	*Relationship of severity or chronicity of the presumed offending infection with cancer initiation and severity in human longitudinal studies or in experimental models demonstrate the association of infectious dose with development of cancer (* Chen et al., 2006 *;* Yang et al., 2016 *)*	Organoids, animal models, or clinico-epidemiologic longitudinal studies	
8. Plausibility (*)	*Are there plausible mechanisms for considering a potential role for a microbe*	Knowledge-based; i.e. understanding the physiology of microbial interaction with humans may suggest or support a role in carcinogenesis	
9. Impact of co-Factors (*)	*Microbial carcinogenesis occurs (or is accelerated) in an environment with promotive host factors, such as genetic risk, immunodeficiencies, nutritional status, and/or with environmental factors (including but not limited to known carcinogens) (* Kim et al., 2012 *;* Gargiulo Isacco et al., 2023 *)*	Epidemiologic and laboratory studies in humans and animal models	

Importance or strength the evidence provides is given in stars, with:

***Gold standard evidence. If one of these criteria is met, it highly suggests that the microbial agent is causative,

**Highly suggestive or supportive information for association

*Further investigation required.

+For criterion 4, primate models or non-human primate models are rated *** while mouse models, or *in vitro* models are rated **. Organoid models are an evolving field and need further assessment as to their confirmatory strength.

Context applies in all these criteria as new discoveries are being made, and new technologies are developed. This table is unable to provide all the nuances that may apply. For example, criteria such as epidemiological data are associated with a level of significance.

## Potential microbial triggers for cancer needing further investigation

A variety of microbes are hypothesized to trigger oncogenesis with a number of criteria for causation met (including the plausibility criterion) (**Table 3**), but each needs further investigation. We recognize that our list of hypothesized and potential microbial facilitators is likely incomplete—our intent is to describe those microbial cancer pairs for which research has provided some compelling clues. A few examples are provided below.

**Table 3:** Known and hypothesized links between infectious disease and cancer based on the proposed causation criteria.

Pathogen	Cancer associations	Level of evidence	Criteria met	References
Virus				
Epstein-Barr virus	Lymphomas, nasopharyngeal, head and neck cancer	Confirmed	1, 2, 3, 4, 5, 7, 8, 9	(Young and Murray, 2003; Brady et al., 2007; Carbone and Gloghini, 2018; Burton and Gewurz, 2022; Su et al., 2023)
	Stomach cancer	Medium	1, 2, 3, 5, (7, 8) may be dependent on cofactors	(Young and Murray, 2003; Tavakoli et al., 2020; Jeong et al., 2022; Salnikov et al., 2024)
	Breast cancer, squamous cell carcinomas (oral, conjunctiva)	Hypothesized	(1)	(Núñez-Acurio et al., 2020; Tavakoli et al., 2020; Jeong et al., 2022; Julius et al., 2022; Heawchaiyaphum et al., 2023; Salnikov et al., 2024)
Hepatitis B Virus	Hepatocellular carcinoma	Confirmed	1, 2, 3, 4, 5, 6, 7, 8, 9	(Levrero and Zucman-Rossi, 2016; Zeisel et al., 2021; Flores et al., 2022; Péneau et al., 2022; Cui et al., 2023)
Hepatitis C Virus	Hepatocellular carcinoma	Confirmed	1, 2, 3, 4, (5), 6, 7, 9 mechanism partially understood	(Nash et al., 2010; Hwang et al., 2019; Koike and Tsutsumi, 2021)
	Non-Hodgkin lymphoma	Strong	1, (2, 3, 4, 5), 6, (7), 8	(Tasleem and Sood, 2015; Alkrekshi et al., 2021)
HHV8	Kaposi sarcoma	Confirmed	1, 2, 3, 4, 5, 8, 9	(Akula et al., 2001; Dollard et al., 2010; Dow et al., 2013; Newton et al., 2018; Wan et al., 2024)
HIV**	Cofactor (Kaposi sarcoma, cervical cancer)	Confirmed (as a co-factor)	1, 3, 6, 7, 8, 9	(Liu et al., 2018; Looker et al., 2018; Yarchoan and Uldrick, 2018; Atallah-Yunes et al., 2020)
	Non-AIDS defining (NHL, Hodgkin, liver, lung, anal, head and neck)	Medium (as a co-factor)	1, likely indirect	(Brune et al., 2016; Sigel et al., 2017; Yarchoan and Uldrick, 2018; Khandwala et al., 2021; Navarro et al., 2021; Haas et al., 2022; McGee-Avila et al., 2024)
HPV	Cervical, head and neck, oral, throat, cervical, penile, anal	Confirmed	1, 2, 3, 4, 5, 6, 7, 8, 9	(Williams et al., 2010; Picard et al., 2016; Hoppe-Seyler et al., 2018; Oyervides-Muñoz et al., 2018; ICO/IARC Information Centre on HPV and Cancer, 2023; Jensen et al., 2024)
	Prostate, esophageal, colorectal and breast cancer	Hypothesized	(1, 2, 3), 8	(Ludmir et al., 2015; Hsu et al., 2022; Kudela et al., 2022; Tsydenova et al., 2023)
Merkel cell polyoma virus	Merkel cell carcinoma	Confirmed	1, 2, 4, 5, 9,	(Arora et al., 2012; Yang and You, 2022)
HTLV-1	Leukemia, Lymphomas Acute T-cell Lymphoma	Strong	1, 2, 3, 4, 5, 8 ATL is rare in endemic areas, despite high HTLV-1 prevalence. Potentially cofactor dependent	(Bangham, 2023; Zhi et al., 2020)
Polyoma viruses	Colon cancer	Hypothesized	(1, 2), 8	(Shoraka et al., 2020; Zheng et al., 2022)
Cytomegalovirus	Glioblastoma	Medium	(1, 2) (4, 5) (8, 9)	(Scheurer et al., 2008; Yang et al., 2022a; Guyon et al., 2024; Mercado et al., 2024)
Mouse Mammary Tumor Virus/ Human Mammary Tumor Virus	Breast cancer	Hypothesized	(2), (8) 4 (in mouse models only; not HMTV)	(Lawson and Glenn, 2022; Parisi et al., 2022)
SV40	Mesothelioma, glioblastoma	Hypothesized	(1, 2), (8, 9) 4 (mice)	(Rotondo et al., 2019)
Human Herpes Virus 6	Lymphomas/ glioblastoma	Hypothesized	(1, 2), 8/ 1, (2, 3), 8 ±	(Kiani et al., 2016; Gu et al., 2019; Wells et al., 2019; Chen et al., 2023)
SARS-CoV2	–	Hypothesized	8	(Jahankhani et al., 2023)
Bacteria				
*Helicobactor pylori*	Gastric	Confirmed	1, 2, 3, 4, 5, 6, 7, 8, 9	(Parsonnet et al., 1991; Franco et al., 2005; Salvatori et al., 2023)
	Lung/ Esophageal	Under investigation	(1)/ 1, 6,	(Islami and Kamangar, 2008; Mounika, 2013)
*Fusobacterium nucleatum*	Colorectal	Strong	1, 2, 4, 5, 8, (9)	(Kostic et al., 2013; Ou et al., 2022; Wang and Fang, 2022; Zepeda-Rivera et al., 2024)
*Streptococcus gallolyticus (S. bovis)*	Colon cancer	Strong	1, 2, 4, 5, 8, (9)	(Abdulamir et al., 2011; Kreikemeyer et al., 2018; Eldegla et al., 2021; Şahin et al., 2023; Taylor et al., 2023)
*Salmonella* T*yphi*	Gall bladder	Medium	1, (4, 5), 8	(Nagaraja and Eslick, 2014; Sepe et al., 2020; Upadhayay et al., 2022)
*Chlamydia trachomatis*	Cervical cancer	Medium (co-factor)	1, (2), (4, 5), 8, 9	(Zhu et al., 2016; Challagundla et al., 2023; Gargiulo Isacco et al., 2023)
*Escherichia coli*	Colorectal cancer, UTI leading to bladder cancer,	Medium	1, 4, (5), (8, 9)	(Abd-El-Raouf et al., 2020; Lichtenstern and Lamichhane-Khadka, 2023)
*Peptostreptococcus anaerobius*	Breast cancer, colorectal	Medium (new)	(1, 2), (4) ±	(Tsoi et al., 2017; Long et al., 2019; Gu et al., 2023; Hurst et al., 2024; Liu et al., 2024)
*Microbiome (dysbiosis)*	Colorectal, breast, pancreatic, cervical, blood cancers	Co-factor, therapy modulating	1, 2, (4) many confounders	(Francescone et al., 2014; Koay et al., 2018; Kadosh et al., 2020; Ciernikova et al., 2021; Guo et al., 2021; Akbar et al., 2022; Sohail and Burns, 2023; Pourali et al., 2024)
*Cutibacterium acnes*	Prostate	Hypothesized	(1, 2), 8	(Davidsson et al., 2016, Davidsson et al., 2021; Sayanjali et al., 2016; Zhang et al., 2018)
*Bacteroides fragilis*	Colorectal	Hypothesized	(1, 2), 4, 5, (8)	(Cheng et al., 2020; Scott et al., 2022; Dadgar-Zankbar et al., 2023)
*Mycoplasma*	Lung, breast, ovarian	Hypothesized, co-factor	(1, 2, 5, 8)	(Tantengco et al., 2021; Yacoub et al., 2021; Pang et al., 2023; Benedetti et al., 2024)
Parasites				
*Schistosoma haematobium*	Bladder cancer	Confirmed	1, (2), 3, (4, 5), 6, 7, 8	(Botelho et al., 2009; Zaghloul et al., 2020)
*Clonorchis sinensis*	Cholangiocarcinoma	Confirmed	1, (2), 3, 4, 5, 6, 7, 8	(Shin et al., 2010; Wonid et al., 2019; Chang et al., 2021; Ren et al., 2025)
*Opisthorchis viverrin*	Cholangiocarcinoma	Confirmed	1, (2), 3, 4, 5, 6, 7, 8	(Sripa et al., 2007, Sripa et al., 2012; Perakanya et al., 2022)
*Schistosoma mansoni*	Hepatocellular	Medium (co-factor)	(1, 2, 3, 4, 5, 6), 7, 8	(Toda et al., 2015; Roderfeld et al., 2020; von Bülow et al., 2021)
*Schistosoma japonicum*	Hepatocellular	Medium (co-factor)	(1, 2, 3, 4, 5, 6), 7, 8	(Liu et al., 2022; Kato et al., 2024; Sheng et al., 2024)
*Entamoeba histolytica*	Colorectal cancer	Hypothesized	(1, 2), 8	(Ankri, 2015; Goel et al., 2018; Haghighi et al., 2022)
*Trichomonas vaginalis*	Cervical, prostate	Hypothesized	(1, 2), 8	(Zhang et al., 2023)
*Toxoplasma gondii*	Glioblastoma	Hypothesized	(1), (5), 8	(Zhou et al., 2019; Hodge et al., 2021; Abdollahi et al., 2022; Jung et al., 2022)
Fasciola hepatica	Liver cirrhosis/cholangiocarcinoma	Hypothesized	(1), 8	(Machicado et al., 2016; Tanabe et al., 2024)
Helminths	(chronic inflammation), leukemia, liver cancer	Hypothesized	8	(Oikonomopoulou et al., 2016; Pastille et al., 2017; Sava et al., 2020; Berriel et al., 2021)
Fungi				
*Aspergillus flavus, Aspergillus parasiticus* (aflatoxin)	Liver	Confirmed	1, (2), 3, 4, 5, 6, 7, 8, 9	(Zhu et al., 2021; Jin et al., 2023; Khan et al., 2024)
	Lung	Hypothesized	(1), (5), 8	(Cui et al., 2015; Kang et al., 2020)
*Candida albicans*	Oral, esophageal, anogenital cancer	Hypothesized	(1, 2), 8	(Di Cosola et al., 2021; Wang et al., 2023a, Wang et al., 2023b; Malavika et al., 2025; Guo et al., 2025)
Fusarium species (mycotoxin)	Esophageal, renal, liver, testicular, lung cancer	Hypothesized	1 for esophageal,	(Dragan et al., 2001; Ekwomadu et al., 2022; Khan et al., 2024)

Criteria in parenthesis (-) are inconsistent across studies or show contradicting evidence.

± relatively new findings, or new link between pathogen and cancer

**HIV is often synergistic and required; however, many of these cancers have separate primary pathogenic causes (such as cervical cancer and HPV). Under non-AIDS defining cancers, we mention those, which may be dependent on HIV associated inflammatory processes, and not necessarily immune suppression. Exposure to similar risk factors (as with HBV infection and Hepatocellular cancer) may contribute to the increases a confounder in epidemiological risk; therefore we have classified HIV as a co-factor. confirmed even though it is not implicated directly in these

Scientific judgment is required to evaluate the strength of the evidence for each criterion, which cannot be captured in categorizing these cancers. However, we propose to use these criteria to determine which infectious disease may be investigated further and is likely to show an associative or causal link. We have classified as follows.

Confirmed= Meets most criteria including one (***) and shows reproducibility across labs, and trials. The confirmed diseases described fit all but 1 or 2 criteria. These also meet consistent reproducibility: i.e. confirmation across independent laboratories, optimally using varied methodologies or models, replication in diverse human populations and geographic settings, concordance between *in vitro*, animal, and human studies.

Strong evidence = meets >4 criteria among proposed microbial oncogenesis criteria (including one ***).

Medium evidence = 2 or 3 criteria met with other criteria not evaluated or not evaluated optimally, inconsistent results or not conclusive results. For example, CMV, as a trigger for glioblastoma, has inconsistent results for criteria 1,2, and for criteria 4,5,8,9, the experimental evidence is not conclusive.

Hypothesized = meets plausibility criterion or one other criterion with either conflicting or suboptimal data within other criteria.

### A virus originally found in mice

A retrovirus known as mouse mammary tumor virus (MMTV) has been shown to cause mammary tumors in rodents; multiple studies have shown that human mammary tumor virus (HMTV), which is 90-95% homologous to MMTV is present more often in human breast cancer tissue when compared with healthy breast tissue (Lawson and Glenn, 2022; Parisi et al., 2022). Using our proposed criteria for causation, HMTV/MMTV meets criterion 4 (with oncogenesis demonstrated in an animal model), and partially meets criterion 2 (Consistent detection of the microbe - virus, bacterium, fungus, or parasite- in cancer tissues compared to healthy controls) with consistent findings in some laboratories, but not across all geographies. When detected, it is not clear whether the virus is part of the causation pathway or whether breast cancer tissue is simply conducive to colonization with the virus (Lawson and Glenn, 2022). Infections with viruses like MMTV may induce signaling alterations which causes negative effects only under certain conditions. Microbiome characteristics might determine such a conducive environment. Recent studies have correlated gut microbiome features with breast cancer stages and progression, although a causative link has also not been established (Lee et al., 2023; Sohail and Burns, 2023; Viswanathan et al., 2023).

### Cytomegalovirus

CMV is highly prevalent in SSA with a pooled prevalence of 81.9% (55–97%) (Bates and Brantsaeter, 2016). The potential oncogenicity of CMV has long been a focus for study. CMV proteins and nucleic acids have been found (meeting Criteria 5d and 5e [**Tables 2**, **3**] in some, but not all, studies) within glioblastoma multiforme tumors suggesting that the virus may play a role in facilitating oncogenic transformation or progression of glioblastomas (Scheurer et al., 2008; Yang et al., 2022a; Mercado et al., 2024). Human astrocytes infected with CMV have formed glioblastoma-like cancers in mice models (Guyon et al., 2024) (Criterion 2) and infection with CMV leads to poorer prognosis for glioblastoma (Criterion 6); targeting CMV infected cells has shown potential for glioblastoma therapeutics (Yang et al., 2022a; Mercado et al., 2024). While the association has not been conclusively confirmed, potential mechanisms include chronic inflammation, immune evasion or modulation, and viral gene expression. A variety of characteristics of CMV infection might contribute to cellular transformation to cancer: induction of expression of pro-angiogenic factors, interaction with oncogenic signaling pathways, such as the phosphatidylinositol 3-kinase (PI3K)/Akt pathway, which is often dysregulated in cancers, and epigenetic changes like DNA methylation and histone changes, which can contribute to tumorigenesis (Chan et al., 2010). Among the cancers contributing to substantial burden in Africa, some studies have suggested that CMV may have a role in breast, prostate and colorectal cancers, among others, presumably with similar mechanisms that have been hypothesized for a putative role with glioblastomas (Yu et al., 2023a).

### Polyomavirus

Polyomaviruses have been linked to colon cancers, with JCPyV and BK polyoma viruses showing the strongest causal links (Gorish et al., 2019; Shoraka et al., 2020). Although the majority of humans are latent carriers for these viruses and no comprehensive SSA data was found, immunosuppression either through disease or natural immune senescence can lead to reactivation. Higher levels of JCPyV and BK are found in colon cancer tissues (Criterion 2), as well as in solid B cell leukemia, when compared with non-cancerous tissue, and have been identified in many other cancers (Gorish et al., 2019; Loutfy et al., 2017). Injection of T antigen derived from JCPyV in mice was shown to cause a variety of cancers including neural and breast and hepatocellular cancers (Criterion 4) (Zheng et al., 2022). Similarly, Merkel Cell polyoma virus shows high prevalence in skin cancer tissues (Klufah et al., 2021).

### Fusobacterium nucleatum

A variety of studies have suggested that the oral anaerobic bacterium *F. nucleatum* may have a role in initiating oncogenic transformation in colonic and rectal cells (Kostic et al., 2013). While more study is needed to confirm this potential association, especially within African settings, there is evidence that colorectal cancer cells when colonized with specific subclades of *F. nucleatum*, may increase the potential for local tumor spread and metastasis (Kostic et al., 2013; Rubinstein et al., 2013; Bullman et al., 2017; Ou et al., 2022; Wang and Fang, 2022; Zepeda-Rivera et al., 2024). We further discuss this possibility within the section on microbiomes.

### Cutibacterium acnes

C. acnes (a skin commensal, implicated in superficial skin infections, especially acne vulgaris) has been shown to colonize the prostate, resulting in chronic inflammation, which appears to be a pivotal factor for prostate cancer (Davidsson et al., 2021; Goldstein and Mascitelli, 2024). Studies have demonstrated the presence of *C. acne* in prostate cancer specimens in much higher proportions than in non-cancerous prostate tissue (Criterion 2) (Kakegawa et al., 2017). Furthermore, cohort epidemiologic studies have shown that severe acne during adolescence is associated with a higher likelihood of prostate cancer later in life (Criterion 1) (Zhang et al., 2018). To demonstrate causation, future studies must evaluate and reproducibly confirm other criteria for causation, as well as validate the epidemiologic evidence. Investigations carried out thus far, have yielded inconsistent results (Drott et al., 2010; Sayanjali et al., 2016). Furthermore, existing data are from European studies with a lack of data from Africa, where prostate cancer appears to occur at an earlier age and can be more aggressive (Davidsson et al., 2016; Janivara et al., 2024). That gap needs to be filled to drive further research that could lead to preventive approaches, assuming a triggering role was established.

### Salmonella Typhi

*S* T*yphi* can colonize the gall bladder following symptomatic typhoid fever or asymptomatic systemic infection, resulting in chronic carriage in 1-4% of patients acutely infected. In SSA there are 1.2 million acute typhoid fever cases annually; however, it is unclear how many of these infections lead to chronic carriage (Kim et al., 2024). Moreover, there are distinct genotypes in SSA that have a higher propensity for invasive disease and antibiotic resistance — both factors could influence potential for chronic infection (Kingsley et al., 2009). Gall bladder colonization results in chronic inflammation directly and by stimulated the formation of gallstones. A meta-analysis of 17 studies suggested that chronic *S* T*yphi* infection of the gall bladder is associated with gallbladder cancer (Criterion 1) (Nagaraja and Eslick, 2014; Upadhayay et al., 2022). *S Typhi* has a high prevalence in sub-Saharan Africa with a substantial proportion of infections being undiagnosed or untreated. This is coupled with the increasing incidence of multidrug resistance S Typhi, and low vaccination levels (Kariuki and Onsare, 2024; Khan et al., 2022).

### SARS-CoV-2

While SARS-CoV-2 has not yet been linked to specific cancers (Jahankhani et al., 2023), there has not been sufficient follow-up period to observe an effect. However, this virus has distinct infection-associated patterns which may contribute to being an oncogenic virus. For instance, SARS-CoV-2 causes RAAS (renin-angiotensin-aldosterone system) pathway dysregulation, induces degradation of the tumor repressor retinoblastoma protein, via nsp15 and P53 via nsp3, affects cell cycle through among others nsp7, interferes with DNA methylation through NSP8, and generates reactive oxygen species (ROS), all common pathways involved in oncogenesis, making a link to cancer plausible (Criterion 9) (Jahankhani et al., 2023). Evidence of prolonged, persistence of replicating SARS CoV2 in tissues (Yang et al., 2024) raises a potential for chronic inflammation which may also increase a risk of cancer formation. Longitudinal cohorts, such as the Rotterdam study, maintained over time may provide insight into the role of infectious triggers including SARS-CoV2 in oncogenesis (Ikram et al., 2020; Sijtsma et al., 2022).

### Considering a role for fungi

Candida has been observed in colorectal cancer tissue samples and is associated with decreased survival and metastatic disease in colon cancer. Similarly, Blastomyces is has been detected in lung cancer tumor tissues (Dohlman et al., 2022). However, for these and other examples of fungal colonization, association but no direct causal relationships have been established. Fungal colonization has been suggested to drive carcinogenesis through immune recruitment of TH2 cells in pancreatic and esophageal cancer. Pathogenic infections of Candida species correlate with a higher oral cancer incidence (Chung et al., 2017; Di Cosola et al., 2021). These associations may partially be a consequence of the lower levels of immunity in these individuals (increasing risk for colonization) or could indicate synergistic relationships that promote both cancer and fungal colonization.

Fungi play a demonstrable role in hepatocellular cancer, however in a more indirect way, where they (Aspergillus flavus and Aspergillus parasiticus mainly) infect food sources such as maize and grains, imparting high levels of hepatotoxic aflatoxins which in turn directly contribute to oncogenesis (Cui et al., 2015; Jin et al., 2023; Yu et al., 2023b). This is of particular importance in some regions of SSA (especially west Africa and parts of east Africa) where food storage conditions combined with high heat and humidity contribute to a high aflatoxin burden in staple foods (Falade et al., 2022). Other mycotoxins have been studied such as Ochratoxin A, produced by aspergillus or T-2 and Zearalenone, both fusarium toxins; these link to nephropathies and potentially neurological disorders such as Parkinsons and dementia (Khan et al., 2024). However, these toxins were linked to various cancers (esophageal, kidney, colon, urinary tract, gastrointestinal, uterine, breast) in animal and in *in vitro* models (Claeys et al., 2020; Ekwomadu et al., 2021, Ekwomadu et al., 2022; Khan et al., 2024). For example, fumonisins have been linked to esophageal cancer with recent studies suggesting that it affects PI3K/Akt pathway in human esophageal cells (Yu et al., 2021). Nonetheless, clear epidemiological evidence for these links has not been established (Claeys et al., 2020; Ekwomadu et al., 2022).

### The microbiome

In healthy individuals the gut microbiota, consisting of bacteria, bacteriophages, viruses, archaea, and fungi, play a role in immune regulation through presentation of short chain fatty amino acids (SCFAs) (Mann et al., 2024). *Firmicutes* and *Bifidobacteriaceae* species present these SCFA’s which are taken up by the intestinal cells and regulate the pro-inflammatory cytokines, TNFa IL12 and IL6. Moreover, the microbiome also trains the immune system and inhibits the growth of pathogenic biota (such as *Enterobacteriaceae*) and the development of pathobionts. Pathobionts are microbes that under normal circumstances do not cause disease; however, in the context of cancer or microbiome dysregulation, they become pathogenic (Jochum and Stecher, 2020).

One such pathobiont is *F. nucleatum*, an oral commensal anaerobic bacterium, which as mentioned above, may play an important role in facilitating colorectal cancer incidence and metastasis*. F. nucle*atum colonizes colorectal cancer cells through Fap2, a galactose adhesion hemagglutinin. It produces virulence factors such as FadA, which provides a scaffold for colonization with other bacteria, contributing to dysbiosis, potentially inducing oncogenesis in host cells. FadA and other virulence factors (e.g. AvrA in *Salmonella*) bind to the E cadherin receptor, inducing the *Wnt* signaling pathway, one of the major pathways implicated in colorectal cancer oncogenesis and progression (Kostic et al., 2013; Rubinstein et al., 2013; Bullman et al., 2017; Silva-García et al., 2019). It may enhance colorectal cancer proliferation by upregulation of the *wnt* signaling pathways and metastasis by inducing the expression of CXCL1 and IL-8 which promotes migration and upregulating CCL20 (Ou et al., 2022). *F nucleatum* also induces immune evasion through binding of FapA to immune cells. It is similarly potentially implicated in oral cancers where it enhances proliferation and inhibits cell cycle control mechanisms through p27 (Chen et al., 2022; Wang and Fang, 2022). Lastly it was shown induce metastases by modulating mitogen-activated protein kinase p38, which is involved in mesenchymal transition (Lin et al., 2016).

While there has been a paucity of data characterizing microbiomes in SSA, recent studies have revealed unique taxa and diversity in both South African and Tanzanian samples (Nobels et al., 2025). Click or tap here to enter text. Some investigations have examined the role of the microbiome in cancer development in SSA, linking cervical microbiome characteristics and cervical cancer, as well as suggesting that changes to the gut microbiome after urbanization may correlate with development of colon cancer (Klein et al., 2020; Come et al., 2021; Yang et al., 2022b; Ramaboli et al., 2024), However, most microbiome research in SSA relies on time-intensive culture-based experiments, compared to the more rapid sequence-based technology applied in higher income settings (Paulo et al., 2023; Ayeni et al., 2024). Substantial knowledge gaps remain regarding links between microbiome characteristics and cancers in SSA, opening the door for prioritizing support for pivotal research on the topic

## Complex interplay between the microbiome and cancer

Disturbances in microbiota may alter metabolic pathways and disrupt homeostasis, leaving vulnerabilities to disease (Francescone et al., 2014). The microbiome’s role in cancer development is complex; both protective and pro-cancer effects, which may be dependent on other external factors, have been demonstrated (Kadosh et al., 2020; Akbar et al., 2022).

Cancer associated mutations can also have different outcomes depending on the microbiotic background. This has been shown in p53 mutations, which can cause either oncogenesis or tumor repression depending on the microenvironment. The presence of gallic-acid-producing bacteria in the distal gut induced cancer in mice, while its presence in the proximal gut provided protective effects due to *wnt* signaling inhibition (Kadosh et al., 2020). Similar supporting roles have been found in Kras and p53 mouse models, which in the absence of microbiota in the lung could not cause lung cancer (Jin et al., 2019).

Similarly, bacteria can acquire additional proteins which change them into pathobionts, such as, E coli, when expressing colibactin. This mutagen is found to trigger mutational signatures related to oral squamous cell carcinoma (Boot et al., 2020). Similar mutational signatures which are oncogenic have also been found in colorectal cancer and could indicate a common mutagen in these groups of cancers (Boot et al., 2020; Koh et al., 2021; Cornish et al., 2023).

Dysregulation of the microbiome may also impact treatment as outcomes after hematopoietic stem cell transplantation depend strongly on regulation of inflammation and barrier integrity. The microbiome can modulate the effects of radiotherapy where treatment of dysbiosis with vancomycin can enhance radiotherapy efficacy in melanoma and lung cancer mice models (Ciernikova et al., 2021; Viswanathan et al., 2023; Zhao et al., 2023). The latter may be a cause of complacency which is discussed below. The role of the microbiome in cancer was recently reviewed (Nobels et al., 2025).

### The microbiome as an inconsequential bystander

While some bacteria may play a causative role in cancer, contributing to immune evasion and cancer progression, dysregulation is often a consequence of opportunistic infections, indicating inconsequential presence of bacteria within the microbiome, rather than causation. There is a host of studies where non-commensal or dysbiotic bacteria such as *Salmonella* or *Helicobacter* bacteria are found in tumor tissue (Zhao et al., 2022; Zhu et al., 2022; Schorr et al., 2023). An analysis determined that for most solid tumors, 10^5^ to 10^6^ bacteria are present per palpable 1-cm^3^ tumor, which represents 34 bacterial cells per 5000 cancer cells. The levels of these bacteria are therefore generally low, which complicates analyses. Even when presence is established, presence is not sufficient to indicate causative or synergistic relationships. Indeed, studying the interaction between microbiota and cancer needs careful consideration as demonstrated by a recent re-analysis of links between pancreatic cancer and the microbiome, initially suggesting, then refuting an oncogenic role for microbiota (Guo et al., 2021; Fletcher et al., 2023; Eckhoff et al., 2024; Pourali et al., 2024). The authors argue that the low-biomass of human tissue specimens increases the risk for errors, including distinguishing between low-biomass microbial communities and contamination introduced during sample collection, and errors made during processing, and sequencing. Therefore, PCR confirmed presence and characterizations of the microbiome, cannot not indicate that these organisms were viable in this tissue, nor can determine whether they were causative or bystanders. To form a better understanding of this complex interplay, standardized methods are needed for generating and analyzing microbiome sequencing data to enhance the reproducibility of results across different studies (Aykut et al., 2019; Fletcher et al., 2023).

HIV has also been associated with significant changes in the microbiome. Upon infection, there is rapid spread throughout the lymph system of the gut through mechanisms of cell-to-cell transmission. HIV uses virological synapses to spread throughout the entire CD4 T cell network causing massive cell death and local immune dysregulation. These also result in permanent disruptions in the epithelium of the gut. Even when anti-retroviral therapy (ART) is given early during the course of HIV infection, the gut based immune system is not fully restored and the damage to the epithelial damage causes long lasting dysbiosis and microbial translocation (Zicari et al., 2019; Govindaraj et al., 2023). Microbiota associated with HIV infection are similar to those associated with other inflammatory diseases, such as inflammatory bowel disease. The dysbiosis in HIV may contribute to the continued systemic inflammation (with corollary impacts on cancer risks) observed in HIV, which persists in patients on ART (Serrano-Villar et al., 2017; Herrera et al., 2019).

## Utilizing new approaches to identify novel microbial links with cancer

As opposed to the 1980s, when the associations between HPV and cervical cancer and H. pylori and gastritis and ulcers, initially, and ultimately with gastric cancer (Warren and Marshall, 1983; Parsonnet et al., 1991) were suggested using histopathology and other relatively primitive (by current standards) tools available at the time, new instruments and techniques will likely accelerate the process for identifying previously unrecognized associations For instance, next-generation sequencing (NGS) allows for comprehensive analysis of microbial communities and the identification of novel pathogens in cancer tissues. Assessing genetic material (metagenomics) recovered directly from such as tumor tissue can potentially identify microbes associated with cancers. Proteomics and metabolomics can identify microbial proteins and metabolites in cancer tissues, providing insights into triggers for carcinogenesis. Applying such experimental approaches to longitudinal cohorts, overlying infection status and cancer incidence over time in large populations can yield hypotheses generation to be applied to more focused studies to identify new microbial facilitators and potentiators of cancer.

In addition, prompting artificial intelligence (AI)/large language models trained on all published literature can provide a systematic approach for prioritizing the most likely carcinogenic mediators and mechanisms for study, especially when financial resources are limited. AI models could suggest novel microbial cancer linkages that have not yet been studied or hypothesized, based on aligning oncogenic pathways with microbial pathophysiologies. With such approaches, we propose strict criteria such as ours to steer AI findings. While some models, have advanced beyond pattern recognition to critical thinking, not all have this capacity. In addition, findings models can be limited by CPU power and availability. Thus, caution is needed in applying AI to the complexities of microbial oncogenicity. AI may yield biased conclusions, as it relies on currently available data, which is often sourced from developed nations. Consequently, infectious triggers for cancer, which are much more common in SSA than in areas where most of the data currently exists (i.e. the “global north”) might be overlooked. AI tools may massively accelerate discovery in this field, but will need careful training and coding to correct for biases, and until this is done, such models should be carefully validated (Estiri et al., 2022; Mbunge and Batani, 2023; Mittermaier et al., 2023; Lawsen, 2025; Shojaee et al., 2025).

### Priority areas for discovery

Many of the microbes that have been shown to be oncogenic for specific cancers may cause additional cancers beyond what has already been demonstrated. HPV, implicated in a host of cancers, including cervical, head and neck, anal and penile cancers, may also be associated with prostate (Tsydenova et al., 2023), breast (Kudela et al., 2022), and colorectal (Hsu et al., 2022) cancers. For example, studies have found a predilection for immunohistochemistry-associated HPV presence in prostate cancer tissue when compared with healthy tissue (Zambrano et al., 2002; Lawson and Glenn, 2020). Further research to determine whether there is a facilitative role for HPV in prostate cancer could be considered a priority since, existing tools to prevent HPV infection would be used differently (in boys, perhaps with boosters later in adulthood) and could have dramatic public health benefits, should it be confirmed that a proportion of prostate cancer is triggered by HPV infection and persistence.

Likewise, there are data suggesting that EBV may be associated with breast cancer and gastric adenocarcinoma (Tavakoli et al., 2020; Jeong et al., 2022; Agolli et al., 2023). Determining such relationships could be pivotal for prioritizing EBV vaccine development. In addition to its role as an established trigger for gastric cancer, H. pylori, has been hypothesized to be linked to lower esophageal adenocarcinoma (Islami and Kamangar, 2008). Finding further cancer associations for *H pylori*, could increase the application of resources to utilize the knowledge to develop diagnostic and prevention tools.

Oncogenesis theories must consider “hit and run” cancer mechanisms, where the oncogenic driver may have initiated processes many years ago and now be undetectable; radiation and known mutagen exposure years prior to cancer detection are classic examples, but the concept also applies to microbial oncogenesis *(* Smith and Saveria Campo, 1988 *;* Tommasino, 2017 *)* This is the case for HPV and head and neck cancers, as well as β-HPV and cutaneous cancers, and may also be implicated in the other oncogenic infectious diseases mentioned in this paper *(* Ferreira et al., 2021, Ferreira et al., 2023). Other causal criteria may be fulfilled, but it may not be possible to find histopathological presence in tumor tissue nor persistence of the viral genome *(* Ferreira et al., 2021). The determinant for causation, in a “hit and run” circumstance, may be a pattern of dysregulation, as discussed in this review, that if present, suggest an infectious trigger. Alternatively, microbes producing similar disruption as observed with known microbial cancer pairs (as with HPV and cervical cancer) may provide an indication despite the offending microbe not being present *(* Irrazábal et al., 2014; Muhammad et al., 2019).

## Concluding vision

Discovery of novel microbial-based triggers for oncogenesis and cancer severity will shine a light on feasible pathways to prevent cancer incidence and mortality globally with greatest impact in low-income settings. Such pathways could include vaccine development or modification in use of existing vaccines, as well as new approaches for screening and diagnosis, and other strategies for prevention, and innovative therapies. Machine learning, combined with advances in experimental tools, and multi-disciplinary global collaborations, bringing expertise together across multiple disparate fields of study for innovative approaches, provides the potential a new era for scientific advances in the field of microbial oncogenesis (Breiman et al., 2023). This opportunity should be prioritized because of its consequential potential to lead to products and strategies that will address the massive growing impact and global inequities in cancer burden.
